# Comparing the Performances of Support Vector Machines and Artificial Neural Networks for Predicting Coronary Artery Diseases: A Cross‐Sectional Study

**DOI:** 10.1155/crp/5575296

**Published:** 2026-05-20

**Authors:** Sahar Shariatnia, Abdolhalim Rajabi, Majid Ziaratban, Aref Salehi, Mohammadali Vakili

**Affiliations:** ^1^ Department of Epidemiology and Biostatistics, School of Health, Mashhad University of Medical Sciences, Mashhad, Iran, mums.ac.ir; ^2^ Department of Biostatistics and Epidemiology, Faculty of Health, Golestan University of Medica Science, Gorgan, Iran; ^3^ Health Management and Social Development Research Center, Golestan University of Medical Sciences, Gorgan, Iran, goums.ac.ir; ^4^ Department of Electrical Engineering, Faculty of Engineering, Golestan University, Gorgan, Iran, gu.ac.ir; ^5^ Ischemic Disorders Research Center, Golestan University of Medical Sciences, Gorgan, Iran, goums.ac.ir; ^6^ Health Management and Social Development Research Center, Department of Biostatistics and Epidemiology, Faculty of Health, Golestan University of Medical Sciences, Gorgan, Iran, goums.ac.ir

**Keywords:** artificial neural network (ANN), coronary artery disease, prediction, support vector machines (SVMs)

## Abstract

**Background:**

Coronary artery disease (CAD) is recognized as an inflammatory condition and remains a leading cause of morbidity and mortality worldwide. Cardiovascular disease (CVD), more broadly, is a major contributor to global death and disability. This study aimed to compare the diagnostic performance of various noninvasive techniques for detecting CAD.

**Methods:**

A cross‐sectional study was conducted involving 758 participants, including 508 patients diagnosed with CAD and 250 without the disease. The diagnostic performance of two machine learning models—artificial neural networks (ANNs) and support vector machines (SVMs)—was evaluated. The classification models were assessed using receiver operating characteristic (ROC) curves, sensitivity, specificity, and overall accuracy.

**Results:**

The study included a total of 758 participants. Among them, 250 individuals (33.6% male and 66.4% female) were diagnosed as non‐CAD cases, while 508 participants (64% male, 36% female) were identified as having CAD. The area under the ROC curve (AUC) for CAD prediction was 0.752 (95% CI: 0.682–0.823) using the ANN model and 0.793 (95% CI: 0.733–0.853) using the SVM model. A statistically significant difference was observed between the performance of the two models in predicting CAD (*p* = 0.03), with the SVM model demonstrating superior predictive performance (AUC = 0.793, 95% CI: 0.733–0.853).

**Conclusions:**

SVMs demonstrated superior performance compared to ANNs in predicting the risk of CAD using simple clinical predictors.

## 1. Introduction

Cardiovascular diseases (CVDs) are the leading cause of universal premature death. CVD intimately caused 17.9 million deaths in 2012 [1]. It was also responsible for 347.5 million disability‐adjusted lives (DALYs) in 2015 worldwide [2]. Among these conditions, coronary artery disease (CAD) is the most prevalent and clinically significant form, arising from insufficient oxygenated blood supply to the myocardium due to coronary vessel narrowing. Clinically, CAD is diagnosed when at least one coronary artery exhibits more than 50% stenosis, which may result in angina pectoris or myocardial infarction if left untreated [3, 4]. Risk factors for coronary heart disease include positive family history, smoking, high levels of LDL cholesterol, hypertension, age, and uncontrolled diabetes [4, 5]. Accurate diagnosis of CAD is essential for selecting optimal therapeutic strategies and improving long‐term survival. Conventional diagnostic techniques include electrocardiography (EKG), exercise stress testing, chest radiography, cardiac computed tomography, magnetic resonance imaging (MRI), and coronary angiography. Among these, coronary angiography is considered the gold standard for identifying arterial stenosis [4, 6]. Early and accurate diagnosis of coronary heart disease helps in identifying optimal treatment methods in order to increase the chance of long‐term survival [7]. However, in many developing areas of the world, physicians, diagnostic, or therapeutic techniques may not be available to perform the above process. In addition, failure to diagnose CAD and proper treatments put patients′ health at risk in many cases. Early diagnosis of heart disease can provide ultimate preventive measures such as taking drugs, lifestyle changes, angioplasty, or surgery, which may reduce the progression of the disease and its complications [4, 7, 8]. Therefore, accurate and early diagnosis of heart disease in patients is crucial to reduce the mortality rate and improve their long‐term survival rate.

In recent years, computational intelligence and machine learning (ML) techniques have emerged as promising tools for computer‐aided diagnosis of CVDs. ML‐based systems have been increasingly adopted to support clinical decision‐making by learning complex patterns from clinical data and improving diagnostic accuracy [9]. A wide range of ML algorithms has been applied to CAD prediction, including decision trees [10], artificial neural networks (ANNs) [11], support vector machines (SVMs) [12], fuzzy neural networks [13], particle swarm optimization–based models [14], rotation forest classifiers [15], evolutionary classifiers using principal component analysis [16], K‐star algorithms [17], Bayesian algorithms [18], rule‐based organization methods [19], and fuzzy neural classification techniques [20]. More recent studies have demonstrated that hybrid frameworks combining ML classifiers with optimization or fuzzy‐based strategies can further enhance predictive performance. For example, optimized hybrid models integrating metaheuristic algorithms with intelligent classifiers have shown improved accuracy, robustness, and generalizability in heart disease prediction, speech emotion recognition, remote sensing image retrieval, and quality prediction tasks. These approaches highlight the potential benefits of feature optimization, search space reduction, and parameter tuning in complex classification problems, particularly in medical decision‐support systems [21–24].

Despite these advances, several technical and practical gaps remain in existing CAD prediction studies. Many proposed models rely on limited or highly curated datasets, which restricts their applicability to real‐world clinical environments. Additionally, a considerable number of studies emphasize overall classification accuracy while underreporting clinically critical metrics such as sensitivity, specificity, and the area under the receiver operating characteristic curve (AUC). Furthermore, systematic comparisons between commonly used ML models under consistent evaluation criteria are still scarce. These limitations underscore the need for robust modeling approaches and comprehensive performance evaluation to support reliable early diagnosis of CAD using real clinical data.

Therefore, the present cross‐sectional study develops and systematically compares ANNs trained via error backpropagation and SVMs for the prediction of CAD. Both models are implemented under uniform training and validation conditions and evaluated using accuracy, sensitivity, specificity, and AUC to ensure clinically relevant performance assessment. By providing a rigorous head‐to‐head comparison on real clinical data, this study aims to identify the more effective predictive approach and contribute evidence to guide the selection of ML models in computer‐aided CAD diagnosis. The main contributions of this study are as follows:

• Development and implementation of ANN and SVM models for CAD prediction using real‐world clinical data.
• Comprehensive assessment using clinically meaningful performance metrics, including accuracy, sensitivity, specificity, and AUC.
• Identification of the most effective predictive model to support early detection and informed clinical decision‐making in CAD.

## 2. Methodology

This cross‐sectional study was conducted on patients with suspected CVD who were referred to Fatemeh Zahra Teaching Hospital in Sari, Iran, and underwent coronary angiography. The indications for angiography were based on clinical criteria, including chest pain, chronic coronary syndrome, or acute coronary syndromes such as myocardial infarction, with or without ST‐segment elevation. Demographic, clinical, and laboratory data from 758 individuals were collected to assess the presence of CAD. The primary outcome variable was the presence or absence of CAD, determined based on the cardiologists’ clinical judgment in conjunction with angiographic findings. Details regarding sample size calculation, diagnostic criteria for CAD, collected variables, and patient inclusion criteria have been previously described in a published study [25].

### 2.1. Statistical Methods

#### 2.1.1. ANN Model

The ANN model is a computer structure based on modern neurobiological research, reflecting human brain characteristics. The ANN uses training and learning methods to compare each neuron’s actual output with the expected output to estimate the possible differences between them. Then, depending on the direction of reducing error, each connection weight is modified from the output layer through each hidden layer, layer by layer, and finally each return to the input layer. Thus, the accuracy of input pattern recognition is constantly improved, which can be used to predict the probability of occurrence [26].

Most of the ANN training algorithms use the gradient of the function to determine how to adjust the weights to minimize performance. The gradient is determined using a technique called backpropagation, which involves performing computations backwards through the network. One iteration of this algorithm can be written as

(1) xk+1=xk−αkgk,

where *x*_*k*_ is a vector of current weights and biases, *g*_*k*_ is the current gradient, and *α*_*k*_ is the learning rate. In a Quasi‐Newton method (or secant), an approximate Hessian matrix is updated at each iteration of the algorithm. The update is computed as a function of the gradient. The one‐step secant (OSS) method is an attempt to bridge the gap between the computational complexity of conjugate gradient algorithms and the storage and computation in each iteration requirement in the Quasi‐Newton algorithm. This algorithm does not store the complete Hessian matrix; it assumes that at each iteration the previous Hessian was the identity matrix. The Lavenberg–Marquardt algorithm was designed to approach second‐order training speed without having to compute the Hessian matrix. When the performance function has the form of a sum of squares, then the Hessian matrix can be approximated and the gradient can be computed as in the following equation:

(2) H=JTJ,g=JTe,

where *J* is a Jacobian matrix, which contains first‐order derivatives of the network errors with respect to the weights and biases, and *e* is a vector of network errors. The Jacobian matrix can be computed through a standard backpropagation technique that is much less complex than computing the Hessian matrix. The LM algorithm uses this approximation to the Hessian matrix in the following Newton‐like update, where *x* represents connection weights:

(3) xk+1=xk−JTJ+μI−1JTe.

When the scalar *μ* is zero, this is just Newton’s method, using the approximate Hessian matrix. When *μ* is large, this becomes gradient descent with a small step size [27]. The Levenberg–Marquardt algorithm was used in this study. In our study, we established an ANN model based on factors related to CAD derived from univariate analysis. In the next step, 70% of the data were used for training and 30% were used for testing the ANN model [28].

#### 2.1.2. SVMs

SVM is one of the main supervised learning algorithms presented by Vapnik within the area of statistical learning theory and structural risk minimization. It has been successfully applied to several classification and forecasting problems [29]. SVM is used in regression, classification, clustering, and approximation of functions in which people are separated into two categories [30]. SVM is a powerful method for building classifiers. It allows the prediction of labels from one or more feature vectors by creating a decision boundary between two classes. Known as the hyperplane, this boundary is designated to be as far away as possible from the closest data points from each class. The closest points are called support vectors [31].

In the case that the data can be separated linearly, let us assume that we want to separate the set of points {(*x*_1_, *y*_1_), (*x*_2_, *y*_2_), …, (*x*_*k*_, *y*_*k*_)} where *k* = 1, 2, 3…, l and *y*_*k*_ ∈ {+1, −1} into two classes:

Using the separator $\overset{⟶}{w}\overset{⟶}{x}+b=0$, the data are divided into two spaces. The part of the space above this page has the equation of $\overset{⟶}{w}\overset{⟶}{x}+b\geq 0$ while the part of the space below this separator It has the equation $\overset{⟶}{w}\overset{⟶}{x}+b\leq 0$. The data that apply to the equation $\overset{⟶}{w}\overset{⟶}{x}+b\leq -1$ have the label *y*_*i*_ = −1 while the data that apply to the equation $\overset{⟶}{w}\overset{⟶}{x}+b\geq 1$ have the label *y*_*i*_ = 1. Among the separators that exist, the best separator is the one that creates a wider margin between two pages. Finally, we obtained the maximum value from the following relationship:

(4) fx=wx⟶+b=∑k=1tykαk<xk⟶.x>+b.

In the above equation, *α*_*k*_ is the Lagrange coefficients, *x*_*k*_ is the independent input vectors, *y*_*k*_ is the dependent one and *b* is the distance from the constant [32, 33].

### 2.2. Statistical Analysis

Categorical variables were summarized as percentages, while continuous variables were reported as means ± standard deviation. The chi‐square test was used to compare the frequency distribution of categorical variables between the CAD and non‐CAD groups. For continuous variables, the Kolmogorov–Smirnov test indicated a non‐normal distribution. Therefore, the Mann–Whitney *U* test was applied to compare these variables between the two groups. Prior to model development, the dataset was randomly divided into a training set (70%) and a testing set (30%). All variables were standardized using the min–max normalization method, and the transformed data were applied to model training in both subsets. ANN and SVM models were conducted using MATLAB 2017. Predictions from the ANN and SVM models were evaluated using the testing dataset. Model performance was assessed based on accuracy, sensitivity, specificity, and the area under the receiver operating characteristic (ROC) curve to determine the optimal predictive model. The ROC curve serves as a visual representation of model performance, reflecting the balance between sensitivity and specificity in binary classification tasks [34]. A significance level of 0.05 was used for all statistical analyses.

## 3. Results

### 3.1. Sample Characteristics

According to the inclusion and exclusion criteria, a total of 758 subjects with suspected CAD who underwent coronary angiography were considered for modeling. After angiography, 250 (32.98%) subjects came up to be non‐CAD, and 508 (67.02%) subjects were diagnosed with CAD.

The proportion of smoking and illicit drug use was 8.8% and 5.6%, respectively, in the non‐CAD group, compared to 20.3% and 17.3% in the CAD group, indicating a statistically significant difference between the two groups (*p* < 0.001). Additionally, 64% of individuals in the CAD group were male, compared to 33.6% in the non‐CAD group, reflecting a significant difference in gender distribution (*p* < 0.001). The distribution of blood groups showed that blood group O was the most propotion (37.6%), while blood group AB was the least common (6.9%). Rh‐positive antigen prevalence was 91.6% in the non‐CAD group and 93.7% in the CAD group. These differences in blood group and Rh antigen distribution between the two groups were not statistically significant (*p* > 0.05). A history of hypertension was reported in 38.0% of the non‐CAD group and 41.3% of the CAD group, indicating a higher in the CAD group, though the difference was not statistically significant (*p* = 0.37). A family history of heart disease in first‐degree relatives was present in 15.6% of the non‐CAD group and 21.3% of the CAD group, a difference that was also not statistically significant (*p* = 0.06). Similarly, the proportion of diabetes was 22.4% in the non‐CAD group and 25.8% in the CAD group (*p* = 0.30). Alcohol consumption was reported in 1.6% of the non‐CAD group and 3.5% of the CAD group, with no statistically significant difference (*p* = 0.14).

The mean age was significantly higher in the CAD group (61.46 ± 10.88 years) than in the non‐CAD group (54.33 ± 9.97 years) (*p* < 0.001). The mean body mass index (BMI) was 28.97 ± 5.10 in the non‐CAD group and 27.12 ± 11.40 in the CAD group, with a statistically significant difference (*p* < 0.001). Fasting blood glucose levels were significantly higher in the CAD group (131.39 ± 58.95 mg/dL) compared to the non‐CAD group (116.60 ± 45.25 mg/dL) (*p* < 0.001). High‐density lipoprotein (HDL) levels were markedly lower in the CAD group (38.21 ± 8.41 mg/dL) compared to the non‐CAD group (89.40 ± 59.80 mg/dL) (*p* < 0.001). The mean blood urea nitrogen (BUN) level was 17.37 ± 7.06 mg/dL in the CAD group and 15.07 ± 4.84 mg/dL in the non‐CAD group (*p* < 0.001). Serum creatinine was also significantly higher in the CAD group (1.12 ± 0.88 mg/dL) than in the non‐CAD group (0.98 ± 0.23 mg/dL) (*p* < 0.001). Systolic blood pressure was significantly elevated in the CAD group (129.68 ± 18.44 mmHg) compared to the non‐CAD group (123.45 ± 13.84 mmHg) (*p* < 0.001). Diastolic blood pressure was also significantly higher in the CAD group (78.96 ± 11.17 mmHg) than in the non‐CAD group (76.40 ± 8.91 mmHg) (*p* = 0.001). Total cholesterol (TC) levels were 173.62 ± 5.46 mg/dL in the CAD group and 169.14 ± 47.74 mg/dL in the non‐CAD group; this difference was not statistically significant (*p* = 0.33). Triglyceride levels were 156.77 ± 107.20 mg/dL in the CAD group and 171.91 ± 23.16 mg/dL in the non‐CAD group, with no significant difference (*p* = 0.14). Low‐density lipoprotein (LDL) levels were 94.22 ± 32.73 mg/dL in the CAD group and 89.46 ± 15.31 mg/dL in the non‐CAD group, which also did not show a statistically significant difference (*p* = 0.05) (Table 1).

**TABLE 1:** Comparison of the frequency distribution and mean of the studied variables in patients with and without coronary artery disease (CAD).

Variables	Non‐CAD, *N* (%)	CAD *N* (%)	Total *N* (%)	*p* value
Gender				< 0.001^∗^
Female	166 (66.4)	183 (36)	349 (46)	
Male	84 (33.6)	325 (64)	409 (54)	
Smoking				< 0.001^∗^
No	228 (91.2)	405 (79.7)	633 (83.5)	
Yes	22 (8.8)	103 (20.3)	125 (16.5)	
Illicit drug abuse				< 0.001^∗^
No	236 (94.4)	420 (82.7)	656 (86.5)	
Yes	14 (5.6)	88 (17.3)	102 (13.5)	
Blood group				0.842^∗^
A	80 (32)	148 (29.1)	228 (30.1)	
B	64 (25.6)	129 (25.4)	193 (25.5)	
AB	16 (6.4)	36 (7.1)	52 (6.9)	
O	90 (36)	195 (38.4)	285 (37.6)	
Antigen				0.28^∗^
Negative	21 (8.4)	32 (6.3)	53 (7)	
Positive	229 (91.6)	476 (93.7)	705 (93)	
History of blood pressure				0.37^∗^
No	155 (62)	298 (58.7)	453 (59.8)	
Yes	95 (38)	210 (41.3)	305 (40.2)	
Family history of heart disease				0.06^∗^
No	211 (84.4)	400 (78.7)	611 (80.6)	
Yes	39 (15.6)	108 (21.3)	147 (19.4)	
History of diabetes				0.30^∗^
No	194 (77.6)	377 (74.2)	571 (75.3)	
Yes	56 (22.4)	131 (25.8)	187 (24.7)	
Alcohol use				0.14^∗^
No	246 (98.4)	490 (96.5)	736 (97.1)	
Yes	4 (1.6)	18 (3.5)	22 (2.9)	
Age [mean ± SD]	54.32 ± 9.97	61.45 ± 10.88	59.10 ± 11.10	< 0.001^∗∗^
BMI [mean ± SD]	28.97 ± 5.10	27.11 ± 4.10	27.73 ± 4.54	< 0.001^∗∗^
FBS [mean ± SD]	116.60 ± 45.24	131.39 ± 58.94	126.51 ± 55.21	< 0.001^∗∗^
TC [mean ± SD]	169.13 ± 47.74	173.50 ± 62.45	172.06 ± 58.02	0.33^∗∗^
TG [mean ± SD]	171.23 ± 160.91	156.77 ± 107.20	161.54 ± 127.51	0.14^∗∗^
LDL [mean ± SD]	89.45 ± 31.14	94.21 ± 32.73	92.64 ± 32.27	0.05^∗∗^
HDL [mean ± SD]	40.88 ± 8.58	38.20 ± 8.41	39.09 ± 8.55	< 0.001^∗∗^
BUN [mean ± SD]	15.07 ± 4.84	17.36 ± 7.06	16.60 ± 6.50	< 0.001^∗∗^
Cr [mean ± SD]	0.97 ± 0.22	1.11 ± 0.87	1.07 ± 0.73	0.01^∗∗^
Systolic blood pressure [mean ± SD]	123.45 ± 13.83	129.67 ± 18.44	127.62 ± 17.30	< 0.001^∗∗^
Diastolic blood pressure [mean ± SD]	76.40 ± 8.91	78.96 ± 11.16	78.11 ± 10.54	0.001^∗∗^

^∗^Chi‐square test.

^∗∗^Mann–Whitney *U* test.

### 3.2. Risk Factors for CAD

Table 1 shows the related risk factors of CAD from using a univariate logistic regression model based on all samples (*N* = 758). From univariate logistic regression model, we found significantly protective factors for CAD. Those include HDL (OR = 0.96, 95% CI: 0.94–0.98) and BMI (OR = 0.91, 95% CI: 0.88–0.94). There were also risk factors in terms of gender. Men were more likely to suffer from CAD than women (OR = 3.50, 95% CI: 2.55–4.82), age (OR = 1.06, 95% CI: 1.04–1.08), smoking (OR = 2.63, 95% CI: 1.61–4.29), illicit drug abuse (OR = 3.50, 95% CI: 1.96–6.34), FBS (OR = 1.005, 95% CI: 1.002–1.009), BUN (OR = 1.07, 95% CI: 1.04–1.11), creatinine (OR = 6.56, 95% CI: 3.02–14.26), systolic blood pressure (OR = 1.02, 95% CI: 1.01–1.03), and diastolic blood pressure (OR = 1.02, 95% CI: 1.008–1.03).

### 3.3. Performance of Three Prediction Models

**FIGURE 1:**
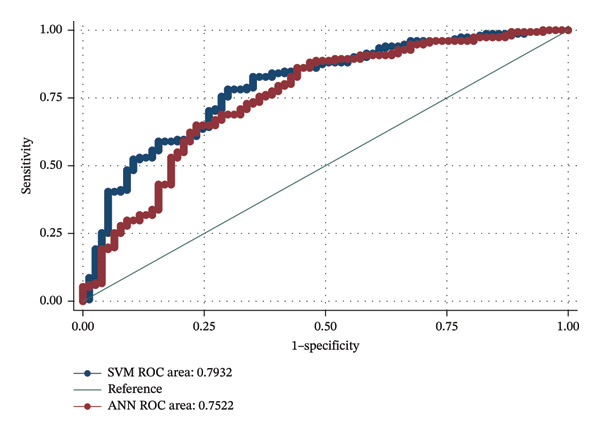
ROC curves of the neural network (ANN) model and support vector machine (SVM) model.

Table 2 compared four prediction models by accuracy, sensitivity, specificity, and AUC. The ANN and SVM models had the same sensitivity, but the specificity of the SVM was extremely low. The AUC of the ANN model in the test set was 0.752 (95% CI: 0.682–0.823), and that of the SVM model in the test set was 0.793 (95% CI: 0.733–0.853). Figure 1 shows the AUC obtained from the test set of the two models. Comparing the AUC of ANN with SVM showed that the SVM model more accurately predicts the risk of an individual suffering from CAD than ANN by 4.1% (*p* value = 0.03) (Figure 1).

**TABLE 2:** The performance of three prediction models on the test set.

Model	Dataset	Accuracy	Sensitivity	Specificity	AUC (95% CI)
ANN	Test set	0.758	0.861 (0.795, 0.912)	0.558 (0.441, 0.672)	0.752 (0.682, 0.823)
SVM	Test set	0.750	0.861 (0.795, 0.912)	0.532 (0.415, 0.647)	0.793 (0.733, 0.853)

Abbreviations: ANN, artificial neural network; SVM, support vector machine.

## 4. Discussion

In the present study, information from 758 individuals who were referred to Fatemeh Zahra (SA) Hospital in Sari, Mazandaran Province, for angiography was used. In order to investigate the performance of ANN, SVM, and LR models for predicting the occurrence of CAD, 20 independent variables including gender, blood group, age, BMI, history of hypertension, family history of heart disease in first‐degree relatives, history of diabetes, smoking status, drug use, alcohol consumption, fasting blood sugar level, TC, triglycerides, high‐density lipoprotein cholesterol (HDL‐C), low‐density lipoprotein cholesterol (LDL‐C), BUN, creatinine level, systolic blood pressure, and diastolic blood pressure were extracted from patients′ records as well as the response variable of angiography results.

Our study showed that the SVM model had significantly better performance in predicting CAD compared to the ANN model. However, the performance difference between the SVM and LR models in predicting CAD risk was negligible, at around 2%, and this difference was not significant. Similarly, the performance of the ANN model did not show a significant difference compared to the LR model.

In general, our study showed that the examined models do not have a significant difference in terms of accuracy index. However, the ANN model is somewhat better than the other two models in terms of accuracy. The study conducted by Ayatollahi et al. [35], aimed at comparing the performance of the SVM and ANN models in predicting CAD, showed that the accuracy of the SVM model is higher than that of the ANN model. Similarly, the study by Saeedbakhsh et al. [36], which aimed at detecting CAD using ML algorithms, showed that the accuracy of SVM was 88.8% and for ANN it was 88.5%. In Kumar et al.’s study [37], the accuracy of SVM was 99.6%, and for ANN it was 100%. Additionally, Shorewala’s study [38] on predicting CHD using risk factors showed that the accuracy rate for the LR model was 71.4% and for the ANN model, it was 73.9%. These results are consistent with our findings that the ANN outperforms LR in terms of performance. Overall, similar to our findings, most studies show that the accuracy index is close for both models; however, generally SVM performs better than ANN in terms of accuracy index.

Our findings indicate that the sensitivity for predicting CAD is higher for SVM and ANN models compared to the LR model, but SVM and ANN models are equal. In Nusinovici et al.’s study [39], which used this model to predict CVD, the sensitivity for the ANN model was 0.76, 0.74 for the LR model, and 0.61 for the SVM model. As evident from our study, similar to Nusinovici et al.’s findings, the sensitivity in the ANN model is higher than that in the LR model. However, contrary to our findings, Nusinovici et al.’s study showed that the ANN model had a higher sensitivity than the SVM model for predicting CVD. Additionally, Kumar et al.’s study [37] demonstrated that the sensitivity of SVM and ANN models was 0.992 and 100, respectively. Furthermore, Saeedbakhsh et al.’s study [36] showed that the sensitivity of SVM and ANN models was 0.574 and 0.582, respectively. These results are similar to our findings and indicate that SVM and ANN models have similar sensitivity indices. In contrast to our study and other text analyses, Ayatollahi et al.’s study [35] showed that the sensitivity of SVM (0.923) was significantly higher than that of ANN (0.88). This difference may be due to the number of variables considered in predicting CAD and sample size in studies.

The results of our study indicate that the SVM model has a higher efficiency in detecting CAD compared to the other two models based on the AUC index. Specifically, the SVM model outperforms the ANN model and shows a significant improvement. Additionally, this model performs better than the LR model, although not statistically significant. A comparison of ANN and LR models based on the AUC index shows that the LR model performs relatively better but not statistically significant. Cheng et al. [40] conducted a study aimed at predicting the risk of coronary artery stenosis in patients with CAD using logistic regression and ANN models. The study showed that the AUC of the ANN model was 0.958, which is inconsistent with our findings. Ing et al. [41] also conducted a study comparing SVMs and logistic regression models for predicting the results of temporal artery biopsy, which showed that the AUC of the SVM model was 0.825 and that of the LR model was 0.827, with no significant difference in performance between them (*p* = 0.86). Ayatollahi et al. [35] compared SVM and ANN models and found that the SVM model performed better than the ANN model in detecting CAD, which is consistent with our findings. Nusinovici et al. [39] also predicted blood pressure levels using SVM, ANN, and LR models, with AUC values of 0.78, 0.775, and 0.77, respectively, which confirms our findings.

We recommend that gender, marital status, history of heart disease, drug abuse, age, BMI, FBS, HDL, LDL, and blood pressure be used as reliable indicators for predicting CAD. In our study, methods were compared using real‐world datasets to provide information about the overall tendency of data structure in the datasets and assist researchers in choosing the best method for solving classification problems. Based on these observations, SVM analysis is superior to ANN and LR in differentiating CAD patients. Therefore, in addition to common noninvasive diagnostic methods, the SVM technique is recommended as a predictive model with acceptable accuracy, sensitivity, and specificity for CAD detection.

### 4.1. Limitations

This study has several limitations. First, it was a cross‐sectional study based on registry data, and no baseline laboratory information was available for patients prior to drug administration. Second, the dataset was collected from a single hospital, which may limit the generalizability of the developed models; therefore, future studies should consider including data from multiple centers. Third, additional patient information—such as clinical symptoms and electrocardiogram (ECG) data—were not available. However, given the objective of this study, the use of commonly recorded clinical features in hospitalized patients was considered adequate. Another limitation is the relatively small sample size, which may affect the robustness of comparisons between predictive models. Finally, the study did not incorporate image‐based parameters, such as coronary CT angiography, which are commonly used in CAD diagnosis and could enhance both the predictive accuracy and clinical applicability of the model.

## 5. Conclusion

Both ANN and SVM demonstrated moderate diagnostic performance. Although sensitivity was comparable, SVM achieved superior discrimination, with an AUC of 0.793 (95% CI: 0.733–0.853) versus 0.752 (95% CI: 0.682–0.823) for ANN, reflecting a significant 4.1% improvement (*p* = 0.03). However, SVM showed lower specificity, indicating a trade‐off between overall discrimination and classification balance.

Overall, SVM provided better global predictive performance for CAD under the present conditions. Nonetheless, model performance is dataset‐dependent and influenced by feature selection and validation strategy. Rigorous evaluation using clinically relevant metrics is essential prior to clinical application, and larger multicenter studies with external validation are needed to confirm generalizability.

**Nomenclature**
CAD: Coronary artery disease
CVD: Cardiovascular disease
ANN: Artificial neural network
LR: Logistic regression
SVMs: Support vector machines
DALYs: Disability‐adjusted lives
MRI: Magnetic resonance imaging
ROC: Receiver operating characteristics
AUC: Area under the ROC curve

## Author Contributions

Sahar Shariatnia and Mohammadali Vakili conceived of the presented idea and developed the methods. Sahar Shariatnia, Mohammadali Vakili, Abdolhalim Rajabi, and Majid Ziaratban carried out the experiment, built the models, wrote the manuscript, and prepared all figures. Aref Salehi and Abdolhalim Rajabi provided the clinical insights. Mohammadali Vakili supervised the project. All authors discussed the results, contributed to the final manuscript, and reviewed the manuscript.

## Funding

This research was supported by Faculty of Health and Vice Chancellor for Research and Technology of Golestan University of Medical Sciences (Grand no. 110374).

## Disclosure

A preprint of this article has previously been published in Research Square [42]. All authors read and approved the final manuscript.

## Ethics Statement

The study protocol was approved by the Ethics Committee of Golestan University of Medical Sciences (No. IR.GOUMS.REC.1398.031). All participants provided informed consent. All methods were carried out in accordance with relevant guidelines and regulations, and a consent form was obtained from all the participants.

## Conflicts of Interest

The authors declare no conflicts of interest.

## Data Availability

The data that support the findings of this study are available from the corresponding author upon reasonable request.
